# 574 Identifying New or Unmanaged Co-Morbidities to Improve Burn Care Management and Establish Care

**DOI:** 10.1093/jbcr/irae036.208

**Published:** 2024-04-17

**Authors:** Desiree Pinto, Lauren T Moffatt, Taryn E Travis, Jeffrey W Shupp, Shawn Tejiram

**Affiliations:** MedStar Washington Hospital Center, Washington, DC; Firefighters’ Burn and Surgical Research Laboratory, Washington, DC; MedStar Washington Hospital Center, Georgetown University School of Medicine, Washington, DC; MedStar Health |Georgetown University School of Medicine, Washington, DC, DC; Georgetown University School of Medicine, Washington, DC; MedStar Washington Hospital Center, Washington, DC; Firefighters’ Burn and Surgical Research Laboratory, Washington, DC; MedStar Washington Hospital Center, Georgetown University School of Medicine, Washington, DC; MedStar Health |Georgetown University School of Medicine, Washington, DC, DC; Georgetown University School of Medicine, Washington, DC; MedStar Washington Hospital Center, Washington, DC; Firefighters’ Burn and Surgical Research Laboratory, Washington, DC; MedStar Washington Hospital Center, Georgetown University School of Medicine, Washington, DC; MedStar Health |Georgetown University School of Medicine, Washington, DC, DC; Georgetown University School of Medicine, Washington, DC; MedStar Washington Hospital Center, Washington, DC; Firefighters’ Burn and Surgical Research Laboratory, Washington, DC; MedStar Washington Hospital Center, Georgetown University School of Medicine, Washington, DC; MedStar Health |Georgetown University School of Medicine, Washington, DC, DC; Georgetown University School of Medicine, Washington, DC; MedStar Washington Hospital Center, Washington, DC; Firefighters’ Burn and Surgical Research Laboratory, Washington, DC; MedStar Washington Hospital Center, Georgetown University School of Medicine, Washington, DC; MedStar Health |Georgetown University School of Medicine, Washington, DC, DC; Georgetown University School of Medicine, Washington, DC

## Abstract

**Introduction:**

Chronic co-morbidities impact the management of burn patients, as well as their short and long-term outcomes. Conversely and cumulatively, burn injury and the resulting hypermetabolic state can also worsen chronic co-morbidities. The prevalence of diagnosed or undiagnosed existing co-morbidities in burn patients is not well characterized. This study aims to identify the prevalence of new and/or unmanaged chronic co-morbidities after burn injury with admission lab work to determine whether opportunities exist in this area to improve burn care management and manage co-morbidity care.

**Methods:**

This retrospective review included all patients over the age of 18 years old admitted to a regional burn center over a 6-month period (Jan- July 2022). Patient demographics, burn injury characteristics, laboratory values for hemoglobin A1c (Ha1c) and screening tests for HIV and acute hepatitis were collected. Undiagnosed diabetes mellitus (DM) was defined as no prior diagnosis/documentation of DM, no prior medication prescribed for glycemic control, and Ha1c ≥ 6.5. Uncontrolled DM was defined as a prior diagnosis/documentation of DM and a Ha1c > 9. New diagnosis of HIV was defined as no prior diagnosis/documentation of HIV, no anti-retroviral medication prescribed, and a positive screening HIV Ab/Ag result. A new diagnosis of hepatitis C was defined as no prior diagnosis/documentation of hepatitis C and a positive result on the acute hepatitis panel.

**Results:**

Of the 268 patients who presented to the hospital for burn injury, 265 patients were included in the analysis. The mean age was 48.5 and 56% were male. Based on insurance status, 50% of patients had Medicaid/Medicare, 33% had private insurance, and 5% had no insurance. Within our population, 2.2% of patients experienced homelessness. Prior to admission, 16.9% of patients had a diagnosis of DM, of which 22% had uncontrolled DM. There were 7 patients (3.2%) newly diagnosed with DM, and about 60% of these patients had Ha1c levels > 9, a level high enough to consider immediate initiation of insulin therapy. Other screening tests identified a new diagnosis of HIV in 4 patients (1.5%) and hepatitis C in 9 patients (4%). For patients previously diagnosed with HIV or hepatitis C, 22% had unmanaged HIV and 75% have never received treatment for hepatitis C.

**Conclusions:**

Burn injury was used as an opportunity to identify patients with new or unmanaged chronic co-morbidities. A high prevalence was seen in our population, indicating a need to continue screening. Identifying co-morbidities impacted treatment management and referral to other providers, including diabetes educators, infectious disease, and primary care physicians.

**Applicability of Research to Practice:**

A screening tool to identify new or unmanaged co-morbidities is easy to implement and has the potential to improve acute and long-term management and outcomes after burn injury.

